# Occurrence of *Corynebacterium striatum* as an emerging antibiotic-resistant nosocomial pathogen in a Tunisian hospital

**DOI:** 10.1038/s41598-017-10081-y

**Published:** 2017-08-28

**Authors:** Sana Alibi, Asma Ferjani, Jalel Boukadida, María Eliecer Cano, Marta Fernández-Martínez, Luis Martínez-Martínez, Jesús Navas

**Affiliations:** 1 Laboratoire de microbiologie-immunologie, unite de recherché “caractérisation génomique des agents infectieux UR12SP34”, CHU Farhat-Hached, Sousse, Tunisia; 2 Faculté des sciences de Bizerte, Université de Carthage, Jarzouna, Tunisia; 3 Servicio de Microbiología, Hospital Universitario Marqués de Valdecilla-IDIVAL, Santander, Spain; 4 Unidad de Gestión Clínica, Hospital Universitario Reina Sofía, Córdoba, Spain; 5 Instituto Maimónides de Investigación Biomédica de Córdoba (IMIBIC), Córdoba, Spain; 6 Departamento de Microbiología, Universidad de Córdoba, Córdoba, Spain; 7 Departamento de Biología Molecular, Universidad de Cantabria, Santander, Spain

## Abstract

*Corynebacterium striatum* is a nosocomial opportunistic pathogen increasingly associated with a wide range of human infections and is often resistant to several antibiotics. We investigated the susceptibility of 63 *C*. *striatum* isolated at the Farhat-Hached hospital, Sousse (Tunisia), during the period 2011–2014, to a panel of 16 compounds belonging to the main clinically relevant classes of antimicrobial agents. All strains were susceptible to vancomycin, linezolid, and daptomycin. Amikacin and gentamicin also showed good activity (MICs_90_ = 1 and 2 mg/L, respectively). High rates of resistance to penicillin (82.5%), clindamycin (79.4%), cefotaxime (60.3%), erythromycin (47.6%), ciprofloxacin (36.5%), moxifloxacin (34.9%), and rifampicin (25.4%) were observed. Fifty-nine (93.7%) out of the 63 isolates showed resistance to at least one compound and 31 (49.2%) were multidrug-resistant. Twenty-nine resistance profiles were distinguished among the 59 resistant *C*. *striatum*. Most of the strains resistant to fluoroquinolones showed a double mutation leading to an amino acid change in positions 87 and 91 in the quinolone resistance-determining region of the *gyrA* gene. The 52 strains resistant to penicillin were positive for the gene *bla*, encoding a class A β-lactamase. Twenty-two PFGE patterns were identified among the 63 *C*. *striatum*, indicating that some clones have spread within the hospital.

## Introduction

*Corynebacterium* species are widely distributed in the environment and in the microbiota of humans and animals. Medically relevant *Corynebacterium* species include *Corynebacterium diphtheriae*, the primary cause of diphtheria, and the non-diphtherial corynebacteria, which are part of the normal flora of the skin and mucous membranes. Nondiphtherial corynebacteria have been frequently dismissed as a contaminant when isolated from clinical materials. However, the role of these bacteria in clinical disease is now more clearly established. *Corynebacterium striatum* is recognized as a true pathogen when isolated in several samples from sterile body sites or from indwelling medical devices^1, 2^. Consideration of whether an isolate represents infection, colonization, or contamination is based upon clinical assessment. A variety of infections have been associated with this bacterium: bacteremia, endocarditis, valvular damage, meningitis, vaginitis and infections of the urinary tract, the respiratory tract, wounds, skin and eye^3–10^.

Susceptibility testing of *C*. *striatum* is necessary to establish a specific therapy. Although initial studies indicated that *C*. *striatum* clinical isolates were susceptible to a wide range of antibiotics^11, 12^, recent reports have shown increased multidrug resistance^13, 14^. Patients suffering underlying diseases and receiving multiple antibiotic courses are at high risk for developing serious opportunistic infections by drug-resistant *C*. *striatum* strains, which can be at the origin of major outbreaks. Thus, several outbreaks of clonal multidrug-resistant *C*. *striatum* have been recently reported^15–17^.

*C*. *striatum*, considered as an emerging pathogen in different countries, has been the most frequently isolated *Corynebacterium* species since 2011 at the Farhat-Hached University (FHU) hospital, Sousse, Tunisia. In this study, we aimed to investigate the susceptibility of the 63 *C*. *striatum* isolated at the FHU hospital during the period 2011–2014 to 16 agents, representative of the main groups of antibiotics, including the most used compounds to treat infections caused by *Corynebacterium spp*. as well as second-line and complementary agents. We describe the high incidence of drug resistance in our *C*. *striatum* and characterize the molecular mechanisms related to resistance to aminoglycosides, compounds of the MLS_B_ group, fluoroquinolones and β-lactams. The clonal relationships among *C*. *striatum* isolates were analysed by Pulsed-field Gel Electrophoresis (PFGE).

## Results

### Antimicrobial susceptibility testing and resistance profiles

Fifty-nine (93.7%) out of the 63 isolates showed resistance to at least one of the 16 tested compounds, whereas the remaining 4 isolates were susceptible. The MIC_50_ and MIC_90_ distributions and the percentage of resistance to the different antibiotics for the 63 *C*. *striatum* included in this study are presented in Table 1.

**Table 1:** Susceptibility of 63 *Corynebacterium striatum* clinical isolates to 16 antimicrobial agents.

Antimicrobial agent	Range (mg/L)	MIC_50_	MIC_90_	Breakpoint			Resistant	Intermediate	Total (%) R
				S	I	R			
Amikacin	0.06–64	0.06	1	≤16	32	≥64	3	0	3 (4.8%)
Gentamicin	0.06–64	0.06	2	≤4	8	≥16	3	1	4 (6.3%)
Kanamicin	0.016–256	0.125	>64	≤16	32	≥64	10	1	11 (17.5%)
Tobramicin	0.06–64	0.06	8	≤4	8	≥16	3	4	7 (11.1%)
Streptomycin	0.125–64	2	>64	≤8		>16	8	2	10 (15.9%)
Erythromycin	0.06–64	0.5	8	≤0.5	1	≥2	24	6	30 (47.6%)
Clindamycin	0.06–64	1	>64	≤0.5	2	≥4	24	26	50 (79.4%)
Doxycicline	0.06–16	0.06	8	≤4	8	≥16	0	11	11 (17.5%)
Ciprofloxacin	0.015–16	0.125	>16	≤1	2	≥4	21	2	23 (36.5%)
Moxifloxacin	0.06–16	0.06	>16	≤0.5	1	≥2	15	7	22 (34.9%)
Penicillin	0.06–64	1	16	≤0.125			52	0	52 (82.5%)
Cefotaxime	0.25–256	2	16	≤1	2	≥4	28	10	38 (60.3%)
Rifampicin	0.015–64	0.015	16	≤1	2	≥4	14	2	16 (25.4%)
Vancomycin	0.06–1	0.25	0.25	≤2			0	0	0
Linezolid	0.015–1	0.25	0.5	≤4		≥8	0	0	0
Daptomycin	0.015–1	0.125	0.25	≤1			0	0	0

Isolates were classified as resistant, intermediate, or susceptible, according to criteria defined by CLSI^38^.

MIC, minimum inhibitory concentration; MIC_50/90_, MIC that inhibits 50% and 90% of the isolates, respectively.

The MIC_90_ values of vancomycin, daptomycin and linezolid were in the range 0.25–0.5 mg/L, and none of the 63 isolates showed resistance against them. Considering the MIC_90_ values, the most active compounds were vancomycin and daptomycin (MIC_90_ = 0.25 mg/L for both compounds) followed by linezolid (MIC_90_ = 0.5 mg/L). Among the 5 aminoglycosides tested, amikacin and gentamicin were the most active compounds (MIC_90_ values of 1 and 2 mg/L, respectively). Only three *C*. *striatum* were resistant to both compounds (MICs > 64 mg/L). Tobramycin was also very active (56 isolates were susceptible). Kanamycin and streptomycin showed low activity against our *C*. *striatum* (MICs_90_ > 64 mg/L). The compounds of the MLS_B_ group were poorly active against our *C*. *striatum* (MICs_90_ of 8 and >64 mg/L for erythromycin and clindamycin, respectively). Rifampicin was poorly active too (MIC_90_ = 16 mg/L). Ciprofloxacin and moxifloxacin also showed low activity against our isolates (MIC_90_ > 16 mg/L for both compounds). A high number of the isolates were resistant to the β-lactams penicillin and cefotaxime (MICs_90_ = 16 mg/L).

**Figure 1:**
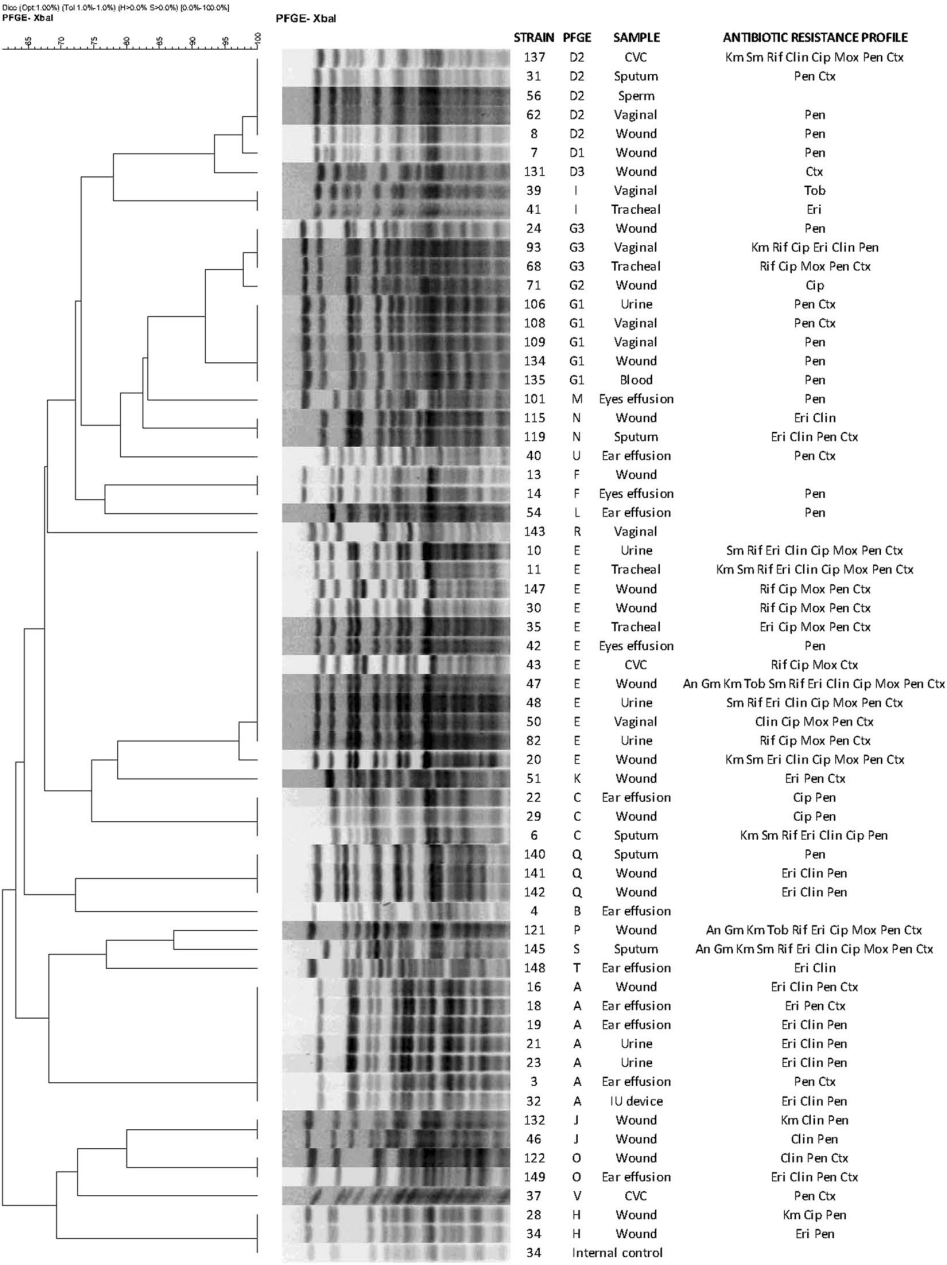
PFGE patterns and antibiotic resistance profiles of the 63 *C*. *striatum*. An: amikacin; Gm: gentamicin; Km: kanamycin; Sm: streptomycin; Tob: tobramycin; Rif: rifampicin; Eri: erythromycin; Clin: clindamycin; Cip: ciprofloxacin; Mox: moxifloxacin; Pen: penicillin; Ctx: cefotaxime.

The 59 *C*. *striatum* resistant to at least one of the tested compounds were divided in 29 resistance profiles (Fig. 1). The most frequently encountered resistance phenotype was penicillin (12 strains), followed by penicillin-cefotaxime and erythromycin-clindamycin-penicillin (6 strains each). Multidrug resistance (MDR), defined as non-susceptibility to at least one agent in three or more antimicrobial categories (as defined for other microorganisms^18^), was observed in 31 (49.2%) strains. Among the 31 MDR isolates, 19 resistance profiles could be distinguished. Eight MDR isolates showed resistance to eight or more compounds, belonging to seven main classes of antibiotics (aminoglycosides, ansamycins, macrolides, lincosamides, fluoroquinolones, penicillins and cephalosporins).

### Molecular detection of resistance genes

The *aph*(*3*′)*-Ic* gene was detected in the 10 strains resistant to kanamycin (MIC > 64 mg/L). However, fourteen strains susceptible to kanamycin were also *aph*(*3*′)*-Ic*-positive. The genes *aph*(*3*″)*-Ib* and *aph*(*6*)*-Id* were detected in 8 isolates, five of them resistant to streptomycin (MICs > 64 mg/L), and the other three showed MICs of streptomycin in the range 1–4 mg/L. The gene *aac*(*3*)*-XI* was found in 7 isolates. Two of them were susceptible to both gentamicin and tobramycin (MICs < 0,06 mg/L) whereas the other five isolates showed MICs of 8 and 16 mg/L for gentamicin and tobramycin, respectively.

Twenty out of the 24 *C*. *striatum* resistant to erythromycin carried the *erm*(*X*) gene. Ten out of 20 carried *erm*(*X*) plus the *erm*(*B*) gene. From the 24 clindamycin-resistant isolates 22 were positive for the gene *erm*(*X*) and 10 out of 22 were also *erm*(*B*)-positive. The *mef*(*A-E*) gene was not found in any strain tested.

The sequences of the QRDR region of the *gyrA* gene of 21 isolates categorized as resistant or intermediate to fluoroquinolones were compared to that of the quinolone-susceptible *C*. *striatum* ATCC 6940 (GenBank accession number AY559038). The relationships between the MICs of ciprofloxacin and moxifloxacin and the mutations in the *gyrA* QRDRs of the 21 isolates are summarized in Table 2. Eleven strains showing MICs = 16 mg/L for these two compounds carried a double mutation at resistance hotspots Ser-87 and Asp-91 (*C*. *striatum* numbering), generating a change from Ser-87 to Phe and another change from Asp-91 to Gly or Ala. In two double mutants showing the same changes the increase of MICs of moxifloxacin was limited to 8 mg/L. Another double mutant with Ala instead of Ser in position 87 and Gly instead of Asp in position 91 showed lower MIC of moxifloxacin (4 mg/L) whereas the MIC of ciprofloxacin was >16 mg/L. Three strains had a mutation at amino acid codon Ser-87 that changes this amino acid for Tyr or Phe. These single mutants showed lower increases of MICs of ciprofloxacin (to 4 or 8 mg/L) and a moderate increase of MICs of moxifloxacin (to 1 mg/L). Two strains showed a replacement in the amino acid codon Asp-91, producing a change from Asp to Gly. The MICs of ciprofloxacin and moxifloxacin for these strains were 2 and 1 mg/L, respectively. Two fluoroquinolone-resistant isolates lacked mutations in their QRDRs.

**Table 2:** Relationship between mutations in the QRDR regions of the *gyrA* gene and the MICs for 21 *C*. *striatum* classified as resistant or intermediate to fluoroquinolones.

Number of strains	Ciprofloxacin		Moxifloxacin			
	MIC (mg/L)	Phenotype	MIC (mg/L)	Phenotype	Ser-87	Asp-91
8	>16	R	>16	R	Phe	Gly
3	>16	R	>16	R	Phe	Ala
1	>16	R	8	R	Phe	Gly
1	>16	R	8	R	Phe	Ala
1	>16	R	4	R	Ala	Gly
1	8	R	1	I	Phe	Asp
2	4	R	1	I	Tyr	Asp
2	2	I	1	I	Ser	Gly
1	8	R	1	I	Ser	Asp
1	4	R	1	I	Ser	Asp

The 52 isolates showing resistance to penicillin were positive for the gene *bla*, encoding a class A β-lactamase. The *C*. *striatum bla* gene encodes a serine hydrolase belonging to the class A β-lactamase family protein. Forty-six out of the 63 *C*. *striatum* were positive for the *ampC* gene, encoding a class C β-lactamase. Forty-two of the 46 *ampC*-positive isolates were penicillin-resistant whereas 4 isolates were sensitive. The *ampC* gene was detected in 22 of the 28 cefotaxime-resistant *C*. *striatum*. The 52 penicillin-resistant isolates had MICs of cefotaxime in the range 1–256 mg/L.

### Analysis of the strain clonal relationship by PFGE

*XbaI* digestion of the 63 *C*. *striatum* isolates revealed 22 distinct PFGE patterns, which were designated from A to V (Fig. 1). On these, PFGE types D and G could be further classified into 3 subtypes. Pulsotype E was predominant (n = 12 isolates) and it was observed in 11 MDR strains, six of which were isolated in the neonatology ward. The remaining 20 MDR clinical isolates belonged to PFGE patterns A (n = 6), C, D2, G3 (n = 2), H, J, K, N, O (n = 2), P, Q (n = 2) and S, indicating that they were unrelated. Other patterns including a significant number of isolates were A (n = 7), D (n = 7) and G (n = 9). Relationship between PFGE patterns and antibiotic resistance profiles could not be established, although the most prevalent PFGE patterns (E and A) included principally MDR isolates. Two pairs of isolates assigned to pulsotype E showed the same resistance profile.

## Discussion

One of the most serious problems related to treatment of the infections caused by *C*. *striatum* is the isolation of multidrug-resistant strains from clinical material and the selection of the appropriate antibiotic therapy for a given type of infection. *C*. *striatum* infections should be treated according to the results of the susceptibility tests. Multiple drug resistance is caused by the interplay of multiple resistance mechanisms those emerge via the acquisition of extraneous resistance determinants or spontaneous mutations. This work highlights the high prevalence of multi-resistant strains and resistance genes among the *C*. *striatum* isolated in a hospital in Tunisia, in particular resistance to aminoglycosides, compounds of the MLS_B_ group, fluoroquinolones, and β-lactams.

The 63 *C*. *striatum* analysed in this study were susceptible to vancomycin and linezolid. In an earlier report using the disk diffusion method, Martinez *et al*.^19^ showed that 31 *C*. *striatum* isolated from clinical samples were susceptible to vancomycin. More recently, Gomila *et al*.^20^ reported that their 52 *C*. *striatum* isolated from patients with chronic obstructive respiratory disease were susceptible to vancomycin too. Vancomycin is still active today and therefore it represents an adequate option for treatment of severe infections caused by *C*. *striatum*. Linezolid has also shown an excellent activity, with MICs routinely below 0.5 mg/L^21^. Gómez-Garcés *et al*.^22^ showed that vancomycin and linezolid were equally active against 30 clinical *C*. *striatum* (MICs_90_ of both compounds = 0.5 mg/L). Linezolid can be considered as an alternative to vancomycin against *C*. *striatum*, although its side effects during the long courses of treatment required for hardware or device-associated infections must be pondered. All our *C*. *striatum* were susceptible to daptomycin (MIC_90_ = 0.25 mg/L). Daptomycin has also been proven to be active against *C*. *striatum*, alone^23^ or combined with rifampicin^24^. Therefore, daptomycin can also be considered as an alternative to vancomycin for treatment of *C*. *striatum* infections, although rapid emergence of high-level daptomycin resistance has been recently reported^25, 26^.

Aminoglycosides are used as complementary antibiotics to treat serious infections caused by diphtheroids. Among aminoglycosides, amikacin and gentamicin showed good activity “*in vitro*” against our *C*. *striatum* (MICs_90_ = 1 and 2 mg/L, respectively). Aminoglycoside resistance occurs through several mechanisms that can coexist simultaneously in the same cell. Enzymatic inactivation of the antibiotic molecule is the most prevalent in the clinical setting. The *aac*(*3*)*-XI* gene, encoding an aminoglycoside 3-N acetyl transferase conferring resistance to gentamicin and tobramycin in *C*. *striatum* ^27^, was not found in our 3 gentamicin-resistant isolates, suggesting that resistance is mediated by another mechanism. Kanamycin and streptomycin are not used in clinical practice in Tunisia but were tested in this study because they are good markers for detecting the presence of the *aph*(*3*′)*-Ic*, *aph*(*3*″)*-Ib* and *aph*(*6*)*-Id* genes. The *aph*(*3*′)*-Ic* gene, encoding an aminoglycoside-O-phosphotransferase implicated in resistance to kanamycin, neomycin, paromomycin, ribostamycin and lividomycin, is part of a larger DNA region containing the *aph*(*3*″)*-Ib* - *aph*(*6*)*-Id* tandem pair of resistance genes conferring streptomycin resistance in *Corynebacterium* spp^28^. Identical aminoglycoside resistance regions were found in the plasmid pTP10 from *C*. *striatum* in close vicinity to the erythromycin and chloramphenicol resistance regions^29^. As expected, the 10 *C*. *striatum* resistant to kanamycin carried the *aph*(*3*′)*-Ic* gene. However, this gene was also detected in strains susceptible to kanamycin, probably due to mutations affecting its coding sequence or its promoter. These results confirm that the *aph*(*3*′)*-Ic* gene is widespread in *Corynebacterium* spp. Resistance to streptomycin in *Corynebacterium* spp. is related to the presence of the tandem of genes *aph*(*3*″)*-Ib* and *aph*(*6*)*-Id*, encoding for aminoglycoside-3″-phosphotransferase [APH (3″)-Ib] and aminoglycoside-6-phosphotransferase [APH (6)-Id], respectively^28^. Five of the 8 isolates resistant to streptomycin carried the *aph*(*3*″)*-Ib* and *aph*(*6*)*-Id* genes, and the remaining three had MICs for streptomycin in the range of 1 to 4 mg/L, probably as a consequence of mutations in the above mentioned genes or in their promoter. Other mechanisms such as active efflux of the antimicrobial and reduced intake into the bacterial cell can contribute to streptomycin resistance in these isolates. Amikacin is eventually prescribed in combinative therapy against severe infections caused by *C*. *striatum* at the FHU hospital. Our results indicate that amikacin is the preferable aminoglycoside for treatment of *C*. *striatum* infections whereas gentamicin could be a valid alternative. However, the occurrence of resistant strains requires continual vigilance.

Erythromycin and clindamycin were inactive against the majority of our *C*. *striatum*, in particular clindamycin, with a MIC_90_ eight times greater than that of erythromycin. This fact confirms the previously reported high prevalence of resistance to compounds of the MLS_B_ group among *Corynebacterium* spp., including *C*. *striatum* ^30^. MLS resistance in *Corynebacterium* spp. is most often mediated by two mechanisms: target-site modification mediated by ribosomal RNA methylases codified by the so-called *erm* genes and active drug-efflux mediated by a membrane efflux pump encoded by the *mef*(*A-E*) gene^31^. Our results confirmed those of previous studies which pointed out that *erm*(*X*) is the most important gene implicated in MLS resistance in *Corynebacterium* spp^29, 32^. For the first time we have detected the *erm*(*B*) gene encoding the ribosomal RNA methylase Erm(B) in *C*. *striatum*. The gene *erm*(*B*) confers high-level resistance to macrolides *in Campylobacter coli* ^33^ and other relevant pathogens but is exceptional in *Corynebacterium* spp^31, 34^. Ten of our strains carried the *erm*(*B*) and *erm*(*X*) genes simultaneously, a characteristic previously reported only in one strain of *C*. *urealyticum* ^31^.

One third of our *C*. *striatum* showed intermediate or high-level resistance to ciprofloxacin and moxifloxacin. Fluoroquinolones have been intensively used at the FHU hospital during the last two decades. Upon antibiotic administration, a selective pressure is created in body organs where fluoroquinolones tend to accumulate. Exposure to fluoroquinolones selects for spontaneous mutants in large bacterial populations, including those that colonize the skin and mucous membranes such as corynebacteria. Thus, fluoroquinolone resistance emerged in clinical isolates of *C*. *striatum* and *C*. *amycolatum* ^35^. Resistance to fluoroquinolones in *Corynebacterium spp*. is caused by mutations in the QRDR of the gyrase gene *gyrA*. In our strains, single amino-acid substitutions in position 87 of the GyrA protein generated ciprofloxacin resistance but double mutations in the *gyrA* gene leading to changes in positions 87 and 91 were necessary for high level resistance to ciprofloxacin and moxifloxacin. In fourteen of the 21 fluoroquinolone-resistant *C*. *striatum*, increases of MICs of ciprofloxacin and moxifloxacin until 16 mg/L were related to a double non-conservative mutation at positions 87 and 91. Sierra *et al*.^35^ also reported double mutations at positions 87 and 91 in the *gyrA* gene of six of their *C*. *striatum*, although the MICs of moxifloxacin for their strains were lower (6–8 mg/L). Five of our strains with single mutations at positions 87 or 91 are still resistant to ciprofloxacin although with lower MICs (in the range 2–8 mg/L) whereas the MICs of moxifloxacin remained at 1 mg/L. Single mutations in the residue Ser-87 or in the residue Asp-91 described by Sierra *et al*.^35^ increased the MICs of ciprofloxacin to 1–6 mg/L, whereas remaining susceptible to moxifloxacin. The higher level of moxifloxacin resistance in our strains suggest the existence of a resistance mechanism additional to mutations in *gyrA*. In two strains no changes in their QRDRs were detected, indicating that resistance was mediated by a different mechanism.

β-lactams are the most broadly used class of antimicrobials. Successful treatments of *C*. *striatum* infections with penicillin^36^ or amoxicillin^37^ have been reported. However, low susceptibility to penicillin and cefotaxime among other β-lactams have been communicated^10, 38^, although the genetic mechanism of resistance has not been characterized so far. Considering the MICs_90_ values (16 mg/L), penicillin and cefotaxime showed the same low activity against our *C*. *striatum*. The fact that the rate of penicillin-resistant strains is higher than that of cefotaxime-resistant strains is explained because the CLSI susceptibility breakpoint for penicillin has recently been dropped from 1 mg/l to 0.125 mg/L^39^. Hydrolysis of β-lactam antibiotics by β-lactamases is the most common mechanism of resistance for this class of antibacterial agents in clinically important bacteria. The β-lactamases are classified by protein sequence in four molecular classes, A, B, C, and D, based on conserved and distinguishing amino acid motifs. Fifty-two of our strains were resistant to penicillin and this resistance was related to the presence of a *bla* gene encoding a class A β-lactamase. The chromosomes of *Corynebacterium jeikeium* K411^40^, *Corynebacterium urealyticum* DSM 7109^41^, and *Corynebacterium resistens* DSM 45100^42^, encode the corresponding counterparts of the *C*. *striatum bla* gene, although it has not been associated with resistance to β-lactams in these species. The *ampC* gene, encoding a class C β-lactamase, was detected in 42 of the 52 penicillin-resistant *C*. *striatum*. The *ampC* genes, which are widely distributed among the Enterobacteriaceae, encode enzymes active on both penicillins and cephalosporins^43^. We show here that two β-lactamase-encoding genes, *bla* and *ampC*, are present in β-lactam-resistant *C*. *striatum*. Our data revealed high resistance rates to β-lactams and a high prevalence of the *bla* and the *ampC* genes among the *C*. *striatum* isolated in our hospital. These data are of value to practitioners, discouraging the use of β-lactam compounds for the treatment of infections caused by *C*. *striatum*.

PFGE is considered the gold standard in epidemiological studies of pathogenic microorganisms, providing important insights into their population structure^44^. Our results showed 22 distinct PFGE patterns from 63 *C*. *striatum* strains. The high diversity of genotypes among the 63 *C*. *striatum* revealed that they are mainly not closely related. Therefore, *C*. *striatum* at the FHU hospital may originate from different lineages and sources instead of expansion of a single clonal lineage. This corresponds to the pathogenic condition of *C*. *striatum* as an opportunistic pathogen that causes occasional disease in predisposed patients. Some PFGE patterns were more frequently isolated, suggesting the existence of a few more prevalent clones. We consider patterns E and A as high-prevalence pulsotypes. Most of *C*. *striatum* assigned to pulsotypes E and A were highly resistant, indicating that the most prevalent clones are highly resistant, as has been previously reported^10, 16^. The fact that many different antibiotic resistance profiles could be distinguished among the strains belonging to a particular PFGE pattern revealed that there was not a single strain but several closely related clones producing sporadic infections.

In conclusion, this study highlights the relevance of *C*. *striatum* as an emerging multidrug-resistant nosocomial pathogen at the FHU hospital. The *C*. *striatum* isolates showed 100% susceptibility to vancomycin, linezolid and daptomycin and high rates of resistance to rifampicin, compounds of the MLS_B_ group, fluoroquinolones and β-lactams. Among the several clones of *C*. *striatum* circulating at the FHU hospital, the most prevalent were the most resistant. Therefore, surveillance of MDR *C*. *striatum* should be continued.

## Methods

### Bacterial strains and growth conditions

During the period 2011–2014, 90 strains recovered from clinical specimens submitted for routine culture to the microbiology laboratory of the FHU hospital were assigned to the genus *Corynebacterium* on the basis of colony morphology, Gram staining, and catalase production. They were isolated in pure culture except the 7 specimens from vaginal swabs, where the *Corynebacterium* were the predominant microorganisms in a poly-microbial culture. In that cases the *Corynebacterium* were considered of clinical significance since they were associated with a strong leukocyte reaction in Gram staining^45^. In all cases the *Corynebacterium* were isolated after two different culture sets. Sixty-three out of the 90 strains were identified as putative *C*. *striatum* using API Coryne V2.0 strips (bioMérieux, Marcy l’Etoile, France). *C*. *striatum* was differentiated from *C*. *amycolatum* by additional phenotypic tests (tyrosine hydrolysis, N-acetylglucosamine assimilation, phenylacetic acid assimilation and susceptibility to the vibriostatic agent O/129). Identification was confirmed by MALDI-TOF using the Vitek MS (bioMérieux) system, in accordance with manufacturer’s instructions. The anatomical sites of specimens from whom the 63 *C*. *striatum* were isolated and the clinical diagnosis for the infected patients are shown in Table 3. All strains were grown on blood agar plates at 37 °C and kept frozen at −80 °C in Brain Heart Infusion broth with 20% glycerol until use.

**Table 3:** Sources of specimens from whom *C*. *striatum* were isolated and clinical diagnosis for the 63 infected patients.

Anatomical site	N° isolates	Diagnosis
Wound	23	Surgical site infection; cellulitis
Vaginal swabs	7	Leucorrhoea
Ear effusion	9	Otitis
Urine	6	Urinary infection; urinary tract catheter colonization
Sputa	5	Chronic obstructive pulmonary disease; pneumonia
Tracheal aspirates	4	Tracheobronchitis
Eyes effusion	3	Congenital infection
CVC tips	3	CVC-exit site colonization
Blood	1	Septicemia
IU Device	1	IU device colonization
Sperm	1	Sterility

CVC: Central Venous Catheter; IU: Intrauterine.

### Antimicrobial susceptibility assays

Antimicrobial susceptibilities were determined by micro-dilution in cation adjusted Muller-Hinton broth and interpreted following Clinical and Laboratory Standards Institute (CLSI) guidelines^39^. Sixteen antimicrobials were tested: amikacin, gentamicin, kanamycin, streptomycin, tobramycin, rifampicin, erythromycin, clindamycin, doxycycline, ciprofloxacin, penicillin, cefotaxime, vancomycin (all of them purchased from Sigma Aldrich, Madrid, Spain), moxifloxacin (Discovery Fine Chemicals, Dorset, United Kingdom), linezolid (Pfizer, Bilbao, Spain) and daptomycin (Cubist, Madrid, Spain). Daptomycin broth was supplemented to 50 mg/L calcium for determinations of susceptibility to that drug. Of note, CLSI susceptible interpretive criteria for penicillin has been recently dropped from 1 mg/l to 0.125 mg/L^39^. MIC values of the antibiotics not considered in CLSI guidelines for *Corynebacterium* spp. (amikacin, tobramycin, kanamycin, moxifloxacin and linezolid) were interpreted in accordance to criteria defined by CLSI for *Staphylococcus aureus* ^46^. Since the CLSI lacks breakpoints of streptomycin for staphylococci we have considered the MIC breakpoints values proposed by the French Society of Microbiology (http://www.sfmmicrobiologie.org/UserFiles/files/casfm/CASFM2013vjuin.pdf) to classify the *C*. *striatum* as susceptible, intermediate or resistant to streptomycin. *Escherichia coli* ATCC 25922 and *Streptococcus pneumoniae* ATCC49619 were used as control strains for susceptibility testing assays.

### Amplification and sequencing of genes related to resistance

The presence of aminoglycoside modifying enzyme (AME) genes common in *Corynebacterium* spp. [*aph*(*3*′)*-Ic*, *aac*(*3*)*-XI*, and the tandem of genes *aph*(*3*″)*-Ib and aph*(*6*)*-Id*] was investigated by PCR. Resistance to compounds of the MLS_B_ group was investigated by amplification of the *erm*(*X*), *erm*(*B*) and *mef*(*A-E*) genes. Resistance to quinolones in *Corynebacterium* spp. is related to point mutations in the sequence of the quinolone resistance-determining region (QRDR) of the *gyrA* gene. Thereby, the QRDR at that gene was amplified and sequenced as previously described^35^. The 63 *C*. *striatum* were analysed for the presence of the *bla* gene, encoding a class A β-lactamase involved in resistance to penicillins and cephalosporins in *Corynebacterium* spp. Primers used to amplify the above mentioned genes are listed in Supplementary Table S1. PCR reactions were performed as previously described^47^. PCR products were purified using the QIAquick PCR Purification kit (Qiagen, Madrid, Spain). Purified DNA was sequenced by Macrogen (Seul, Korea) with the primers outlined in Table S1. Mutations in *gyrA* were identified by aligning sequences of resistant isolates to the sequence of *C*. *striatum* ATCC6940 (GenBank accession number AY559038) using the Clustal W program^47^.

### Pulsed-field Gel Electrophoresis and dendrogram analysis

We obtained *XbaI* macro-restriction patterns of the 63 *C*. *striatum* with a published protocol^48^ and a CHEF-DRIII variable angle system (Bio-Rad, Hercules, California, USA). The PFGE patterns were analysed with Fingerprinting II v4.5 software (Bio-Rad). Each isolate was compared with all other isolates using the Dice similarity coefficient and the unweighted pair Group method with arithmetic means (UPGMA), with 1% of optimization and tolerance. Isolates were classified as indistinguishable if they showed 100% similarity, as closely related subtypes if they showed 95–99% similarity, and as different strains if they showed <95% similarity.

### Ethics statement

This study was performed in accordance with the ethical guidelines of the Declaration of Helsinki (1975). Written informed consent was obtained from each patient from whom samples were taken.

## Electronic supplementary material

Supplementary Information
