# Supplementary material for: Occurrence of Corynebacterium striatum as an emerging antibiotic-resistant nosocomial pathogen in a Tunisian hospital
Source: Sci Rep. 2017 Aug 28;7:9704. doi: 10.1038/s41598-017-10081-y (PMC5573724; doi:10.1038/s41598-017-10081-y)
Supplement: Supplementary file 1 — Supplementary Information [file 41598_2017_10081_MOESM1_ESM.docx]

Occurrence of *Corynebacterium striatum* as an emerging antibiotic-resistant nosocomial pathogen in a Tunisian hospital

Alibi S., Ferjani A., Boukadida J., Cano M.E., Fernández-Martínez M., Martínez-Martínez L. & Navas, J.

**Supplementary Table S1. Primers used in the detection and sequencing of resistance genes.**

| GENE | RELATED RESISTANCE | DNA SEQUENCE (5’-3’) | ANNEALING TEMP | SIZE (bp) | REFERENCES |
| --- | --- | --- | --- | --- | --- |
| *erm(X)* | Erithromycin, Clindamycin | AACCATGATTGTGTTTCTGAACG  ACCAGGAAGCGGTGCCCT | 57ºC | 560 | 1 |
| *erm(B)* | Erithromycin, Clindamycin | GAAAAGGTACTCAACCAAATA  AGTAACGGTACTTAAATTGTTTAC | 52ºC | 639 | 2 |
| *mef(A-E)* | Erithromycin, Clindamycin | GCAAATGGTGTAGGTAAGACAACT  TAAAACAAATGTAGTGTACTA | 52ºC | 399 | 2 |
| *aph(3’)-Ic* | Kanamycin | CGAGCATCAAATGAAACTGC  GCGTTGCCAATGATGTTACAG | 54ºC | 624 | 3 |
| *aph(3’’)-Ib* | Streptomycin | CTTGGTGATAACGGCAATTC  CCAATCGCAGATAGAAGGC | 52ºC | 548 | 4 |
| *aph(6)-Id* | Streptomycin | ATCGTCAAGGGATTGAAACC  GGATCGTAGAACATATTGGC | 50ºC | 509 | 4 |
| *aac(3)-XI* | Gentamicin, Tobramycin | ATGACTACAACCAACGAGATC  CTAAAGCTCCCGGATGTAGAG | 52ºC | 452 | 5 |
| *bla* | Penicillin, Cefotaxime | CAGTCTAGCCACTTCGCCAAT  TGACTGCACGGATGGAGATGG | 55ºC | 808 | This study |
| *ampC* | Penicillin, Cefotaxime | CAATCGGATTCCTGGTCGCT  TGGTTCGCGTGATGTTTTCG | 55ºC | 965 | This study |
| *gyrA* | Ciprofloxacin, Moxifloxacin | GCGGCTACGTAAAGTCC  CCGCCGGAGCCGTTCAT | 60ºC | 337 | 6 |

**REFERENCES**

1. Ortíz-Pérez, A., *et al.* High frequency of macrolide resistance mechanisms in clinical isolates of *Corynebacterium* species. *Microb. Drug.* *Resis.* **16**, 273-277 (2010).
2. Sutcliffe, J., Grebe, T., Tait-Kamradt, A. & Wondrack L*.* Detection of erythromycin-resistant determinants by PCR. *Antimicrob. Agents Chemother.* **40**, 2562-2566 (1996).
3. Miró, E., *et al.* Characterization of aminoglycoside-modifying enzymes in *Enterobacteriaceae* clinical strains and characterization of the plasmids implicated in their difusión. *Microb. Drug Res.* **19**, 94-99 (2012).
4. Gebreyes, W.A. & Altier, C. Molecular characterization of multidrug-resistant *Salmonella enterica* subsp. *enterica* serovar Typhimurium isolates from swine. *J. Clin. Microbiol.* **40**, 2813–2822 (2002).
5. Galimand, M., *et al.* AAC(3)-XI, a new aminoglycoside 3-N-Acetyltransferase from *Corynebacterium striatum*. *Antimicrob. Agents* *Chemother.* **59**, 5647–5653 (2015).
6. Sierra, J.M., Martínez-Martínez, L., Vázquez, F., Giralt, E. & Vila, J*.* Relationship between mutations in the *gyrA* gene and quinolone resistance in clinical isolates of *Corynebacterium striatum* and *Corynebacterium amycolatum*. *Antimicrob. Agents Chemother.* **49**, 1714-1719 (2005).
